# UV-C Peroxymonosulfate Activation for Wastewater Regeneration: Simultaneous Inactivation of Pathogens and Degradation of Contaminants of Emerging Concern

**DOI:** 10.3390/molecules26164890

**Published:** 2021-08-12

**Authors:** Ilaria Berruti, Samira Nahim-Granados, María Jesús Abeledo-Lameiro, Isabel Oller, María Inmaculada Polo-López

**Affiliations:** 1 CIEMAT-Plataforma Solar de Almería, Carretera de Senés Km 4, 04200 Tabernas, Almería, Spain; iberruti@psa.es (I.B.); snahim@psa.es (S.N.-G.); mabeledo@psa.es (M.J.A.-L.); ioller@psa.es (I.O.); 2 CIESOL, Joint Centre of the University of Almería-CIEMAT, 04120 La Cañada, Almería, Spain

**Keywords:** peroxymonosulfate/UV-C, wastewater reclamation, bacteria, contaminants of emerging concern, toxicity

## Abstract

This study explores the capability of Sulfate Radical-based Advanced Oxidation Processes (SR-AOPs) for the simultaneous disinfection and decontamination of urban wastewater. Sulfate and hydroxyl radicals in solution were generated activating peroxymonosulfate (PMS) under UV-C irradiation at pilot plant scale. The efficiency of the process was assessed toward the removal of three CECs (Trimethoprim (TMP), Sulfamethoxazole (SMX), and Diclofenac (DCF)) and three bacteria (*Escherichia coli*, *Enterococcus* spp., and *Pseudomonas* spp.) in actual urban wastewater (UWW), obtaining the optimal value of PMS at 0.5 mmol/L. Under such experimental conditions, bacterial concentration ≤ 10 CFU/100 mL was reached after 15 min of UV-C treatment (0.03 kJ/L of accumulative UV-C radiation) for natural occurring bacteria, no bacterial regrowth was observed after 24 and 48 h, and 80% removal of total CECs was achieved after 12 min (0.03 kJ/L), with a release of sulfate ions far from the limit established in wastewater discharge. Moreover, the inactivation of Ampicillin (AMP), Ciprofloxacin (CPX), and Trimethoprim (TMP) antibiotic-resistant bacteria (ARB) and reduction of target genes (ARGs) were successfully achieved. Finally, a harmful effect toward the receiving aquatic environment was not observed according to *Aliivibrio fischeri* toxicity tests, while a slightly toxic effect toward plant growth (phytotoxicity tests) was detected. As a conclusion, a cost analysis demonstrated that the process could be feasible and a promising alternative to successfully address wastewater reuse challenges.

## 1. Introduction

Nowadays, water scarcity has become a critical issue, and the reuse of reclaimed urban wastewater (UWW) has become a reliable alternative source to address this problem. This practice could have social and economic benefits, enhancing water balance, alleviating the actual stress conditions, and promoting circular economy [1]. To promote the safe water reuse, different challenges need to be successfully addressed, including the addition of an efficient tertiary treatment step in Urban Wastewater Treatment Plants (UWWTPs) to meet water quality limits. The European commission approved in 2020 a regulation on “minimum requirements for water reuse in agricultural irrigation and aquifer recharge” in order to assure the microbiological safety of treated UWW [2]. This regulation includes microbial assessment, of which *E. coli* is the main indicator. However, other pollutants, such as Contaminants of Emerging Concern (CECs) and Antibiotic Resistance Bacteria and Genes (ARB and ARGs), are not yet inserted in the regulation as criteria, but their presence is of concern from the human and environmental health point of view. Therefore, their removal should be investigated for the future implementation of new technologies, reaching a safety UWW reuse [3].

CECs are organic substances that have been detected at low levels in the environment (from ng/L to μg/L) and have the potential to cause an adverse impact on human health and the environment [4]. They include surfactants, flame retardants, pharmaceuticals and personal care products, gasoline additives, pesticides, and endocrine-disrupting compounds (EDCs). Their release into the environment is mainly associated to the continuous discharge of UWW effluents, as they are inefficiently removed by conventional UWWTPs treatments [3]. An additional concern is related to their uptake by crops and accumulation in soil during irrigation with reclaimed water [5]. Moreover, the co-presence of antibiotics, bacteria, and a nutrients-rich environment in UWWTPs promotes the development and the proliferation of ARB and ARGs, posing an additional health risk [6]. It is recognized that ARB infections have higher mortality, and they are estimated to cause 10 million deaths worldwide by year 2050, according to the World Health Organization [7].

Therefore, the upgrade of UWWTPs is required to reach a safer reclaimed water, achieving in addition to the parameters already included in regulations, good CECs, ARB, and ARGs removal performances [8]. The most used tertiary treatment involves chlorination, which is a low-cost method for UWW disinfection, but it is poorly effective in CECs removal and ARGs control, being the main concern of its application related to Disinfection By-Products (DBPs) formation. Ozonation provides good performances for bacteria inactivation and CECs degradation, but the high cost and the generation of brominated DBPs constitute the main drawbacks of this treatment [3]. UV-C is generally recognized as an effective tool for inactivating microorganisms in water, but it is not able to guarantee water safety during storage, due to DNA repair mechanisms and the eventual bacterial regrowth [9].

Consequently, research on highly efficient treatments for both water disinfection and decontamination has been raised in the last decades, including the so-called Advanced Oxidation Processes (AOPs), whose successful results rely on the potential generation of Reactive Oxygen Species (ROS), mainly hydroxyl radicals (^•^OH). Nevertheless, sulfate radical (SO_4_^•−^)-based advanced oxidation processes (SR-AOPs) are gaining attention as promising treatment technologies due to their potential to inactivate a wide range of pathogens and to degrade CECs in different water matrices such as domestic or industrial WW [10,11]. SO_4_^•−^ mainly reacts by electron transfer reactions as the main oxidation mechanism, exhibiting longer half-life, higher redox potential, and higher selectivity than ^•^OH. Moreover, it is consumed by the main water matrix constituents with lower kinetic rates [12].

In SR-AOPs, ROS are generated in solution by the activation of persulfate (PS) or peroxymonosulfate (PMS) by different methods, such as UV-C radiation, transition metals, heat, and alkaline conditions [12,13]. Among these methods, UV-C radiation is one of the most investigated due to its high efficiency supplying the energy needed to cleavage the O-O bond of PMS molecules and generating both, SO_4_^•−^ and ^•^OH.

SR-AOPs based on UV-C radiation have been experimentally proved as efficient in the removal of different CECs using low oxidant dosages (PMS and PS at 0.05–0.5 mmol/L) at very low contact times (4–18 s) [14]. However, the simultaneous disinfection and decontamination of secondary WWTP effluents via SR-AOPs has been poorly investigated [15], with limited information on the process capability to address the problem of ARB and ARGs removal.

Thus, the main goal of this study is the evaluation of PMS/UV-C process performance toward different criteria, such as CECs, bacteria, and antibiotic-resistant targets (ARB and ARGs) removal, toxicity, and costs, to address the urban wastewater reuse challenge and assuring the safe use of reclaimed water. The inactivation of several microbial targets (*E. coli*, *Enterococcus* spp., and *Pseudomonas* spp.) and the degradation of three CECs (Diclofenac (DCF), Sulfamethoxazole (SMX), and Trimethoprim (TMP)) were assessed in simulated and actual secondary effluent from urban wastewater (SUWW and UWW) at pilot plant scale. Then, the occurrence of naturally occurring multidrug (namely ampicillin (AMP), ciprofloxacin (CPX), and trimethoprim (TMP)) resistant *E. coli*, *Enterococcus* spp., and *Pseudomonas* spp. strains and ARGs in UWW was probed, and their removal was investigated. Moreover, a possible toxic effect of residual PMS was evaluated toward *Aliivibrio fischeri* luminescent bacteria (evaluating a possible damage to the receiving aquatic environment after treated effluent discharge) and toward the germination of three seeds, such as *Sorghum saccharatum*, *Lepidium sativum*, and *Sinapis alba* (evaluating phytotoxicity after irrigation with reclaimed UWW). Finally, an economic analysis of the treatment was performed to assess process feasibility.

## 2. Materials and Methods

### 2.1. Water Matrices

Two water matrices were used in this study: (i) Simulated Urban Wastewater (SUWW) and (ii) Actual Urban Wastewater (UWW). SUWW was prepared following a recipe previously reported [16] as a UWW effluent model to guarantee the reproducibility and comparability of results among the different operational conditions. UWW was freshly collected from the effluent of the secondary treatment (conventional activated sludge) of the urban wastewater treatment plant of “El Bobar”, which is located in Almeria, South East of Spain, and several batches were used along the experimental plan. The physicochemical and microbiological characterization of both water matrices are summarized in Table S1 (Supplementary Material).

Physicochemical characterization was done by using a pH meter (multi720, WTW, Weilheim, Germany), conductivity meter (GLP31, CRISON, Barcelona, Spain), turbidimeter (Model 2100N, Hach, Colorado, USA), ion chromatograph (Model 850, Metrohm, Herisau, Switzerland in a column METROSEP C4-250/4.0 (250 mm × 4.0 mm ID)), and Total Organic Carbon (TOC) analyzer (Model 5050, Shimadzu, Kyoto, Japan). The microbiological characterization of UWW was performed following the methodologies described in Section 2.3.

### 2.2. Contaminants of Emerging Concern Quantification

Three common pharmaceutical substances frequently detected in secondary effluents of UWW were used in this study: Trimethoprim (TMP), Diclofenac (DCF), and Sulfamethoxazole (SMX). They were purchased from Sigma-Aldrich with high purity grade (>99%). The initial concentration of each CEC was 100 µg/L, which was obtained by directly diluting a concentrated stock solution of each compound in both water matrices. The CECs quantification was performed by UPLC/UV (Agilent Technologies, Series 1260), following the working conditions described elsewhere [17] to simultaneously detect the three CECs. The limit of quantification (LOQ) was 8, 8, and 20 µg/L for DCF, SMX, and TMP, respectively. The treatment efficiency for CECs removal is established according to 80% removal, value in agreement with the Swiss Water Protection Act, which is the only regulation at the European Level that establishes in Switzerland the requirement of monitoring and removing selected CECs in UWWTPs [18].

### 2.3. Bacteria and ARB Enumeration

*E. coli* O157:H7 (CECT 4972), *E. faecalis* (CECT 5143), and *P. aeruginosa* (CECT 110) provided by the Spanish Culture Collection (CECT) were used as microbial targets in SUWW assays. For this aim, liquid cultures were daily prepared by bacteria incubation at 37 °C and 100 rpm (rotary shaking) for 20 h (stationary concentration of ≈10^9^ CFU/mL) in the specific liquid broth medium for each bacteria according to CECT instructions. Bacterial suspensions obtained were centrifuged (3000 rpm, 15 min), re-suspended in phosphate-buffered saline (PBS) solution, and directly spiked in the reactors to achieve the desired initial concentration (10^6^ CFU/mL). In actual UWW, naturally occurring bacteria (*E. coli*, *Enterococcus* spp., *Pseudomonas* spp., and *Total coliforms*) (occurrence in UWW is shown in Table S1) and AR-bacteria (*E. coli*, *Enterococcus* spp., and *Pseudomonas* spp.) were monitored. 

Water samples from experiments were serially diluted in PBS and enumerated using the standard plate counting method by spreading sample volumes from 50 to 500 µL in selective and specific agar media and its subsequent incubation: ChromoCult Coliform Agar (Merck KGaA, Darmstadt, Germany) 24 h at 37 °C, P. aeruginosa agar (Conda, Pronadisa, Madrid, Spain) 48 h at 35 °C, and Slanetz Bartley agar (Scharlau, Barcelona, Spain) 48 h at 37 °C for *E. coli, Pseudomonas* spp., and *Enterococcus* spp., respectively. When lower concentrations than 10 CFU/mL were expected, the membrane filtration method was also used to attain a detection limit (DL) of 1 CFU/100 mL to fit with the limits established in wastewater reuse guidelines. Then, 100 mL of sample was filtered using a Microfil^®^filtration system (Millipore, Burlington, MA, USA) and cellulose nitrate filters (0.45 μm, Sartorius Stedim, Göttingen, Germany), using similar culture media procedure as described previously.

In addition, ARB were monitored in UWW following the same procedure described above with selective agar media supplemented with three antibiotics according to the respective minimum inhibitory concentration values available in the EUCAST database. Ampicillin (8 mg/L), ciprofloxacin (0.5 mg/L), and trimethoprim (4 mg/L) for *E. coli* and *Pseudomonas* spp.; and ampicillin (8 mg/L), ciprofloxacin (4 mg/L) and trimethoprim (1 mg/L) for *Enterococcus* spp.

### 2.4. Antibiotic-Resistant Genes (ARGs) Detection and Quantification

The *16S rRNA* gene and several ARGs were quantified using real-time quantitative PCR (qPCR). ARGs included genes encoding resistance to quinolone antibiotic classes (*qnrS*), sulphonamides (*sul1*), β-lactams (*bla_TEM_*), cephalosporins (*bla_CTX-M32_*), tetracycline (*tetM*), and class 1 integron integrase (*intI1*), and they were selected due to their occurrence in UWWTPs [8].

UWW samples from El Bobar, UV-C alone test, and PMS/UV-C test at 1 mmol/L were collected and analyzed. A volume of 100 mL of each sample was filtered in duplicated through a 0.2 µm polycarbonate membrane (CHMLAB GROUP S.L., Barcelona, Spain). Total DNA was extracted from the filtering membrane with the DNeasy^®^ PowerWater^®^ Kit (QIAGEN Sciences Inc., Germantown, USA) according to the manufacturer’s indications. The resulting DNA extracts were measured using a NanoDrop^®^ Lite Spectrophotometer (Thermo Scientific, Massachusetts, USA). DNA working solution was stored at −20 °C until they were analyzed.

All genes were analyzed in duplicate by q-PCR using a 7500 Fast Real-Time PCR System (Applied Biosystems, Thermo Fisher Scientific Inc., Massachusetts, USA). The working conditions were done by modifications of reported studies in the literature [19,20], and they are presented in Table S2. Amplification data were analyzed calculating the ratio of each analyzed ARG concerning *16S rRNA* gene (indicator for the total microbial abundance) using the cycle threshold (Ct) value.

### 2.5. UV-C Pilot Plant and Experimental Procedure

A 80 L UV-C (medium pressure lamp with 254 nm peak wavelengths, 230 W) pilot plant was used in this study in batch mode with a flow-rate of 36 L/min and an illuminated volume (V_i_) of 6.21 L. A full description is reported elsewhere [16]. The UV-C chamber accounts with a UV irradiance detector placed in the inner wall of the lamp chamber to monitor the lamp irradiance (W/m^2^) emitted at any time. Irradiance profiles observed under UV-C irradiation in SUWW and UWW are reported in Figure S1. This measurement was used to calculate, according to Equation (1), the UV-C accumulative energy per unit of volume *Q_UV_* (in kJ/L) [16].

(1) $$Q_{UV}\left(\frac{kJ}{L}\right)=Dose\;\left(\frac{kJ}{m^{2}}\right)\times \;A_{i}\left(m^{2}\right)/V_{t}\left(L\right)$$

where *Dose* is obtained by the UV-C lamp emitted irradiance (W/m^2^) multiplied by the illumination time fraction (s), *A_i_* is the illuminated area (0.34 m^2^), and *V_t_* is the total volume (80 L).

Tests procedure was as follows: (i) fill the reactor with the water matrix (SUWW or UWW) and add CECs and bacteria (only for SUWW) to obtain the desired initial concentrations; (ii) homogenization in dark for 15 min; (iii) addition of PMS and collection of the initial sample (time 0 min); (iii) switch-on the UV-C lamp; and (iv) take water samples at regular time intervals for bacterial and CECs quantification. Two replicated experiments of each operational condition were done, and the results of target concentrations detected at any time are presented as the averaged values with their corresponding standard deviation as error.

In addition, water temperature (T) and pH were monitored along the experimental time with a thermometer (Checktemp, Hanna, Gipuzkoa, Spain) and a pH meter (110-K, Horiba Laqua act, Kyoto, Japan). No significant changes in T and pH were observed in any case, remaining constant at values of 28.4 ± 1.3 °C and 8.17 ± 0.04, respectively. Therefore, PMS thermal activation as well as pH effect on CECs and bacteria removal could be discarded in our results.

### 2.6. Kinetic Analysis

All bacterial inactivation profiles observed in this study followed a double log-linear kinetic, each one described by Chick–Watson’s law. This kinetic inactivation model has been described previously for bacterial inactivation by UV-C lamps, and it is attributed to its highly capability to damage cells observed in a very fast first stage (*k*_1_, min^−1^), followed by a second one (*k*_2_, min^−1^) with lower kinetic constant (*k*_1_ >> *k*_2_) [13]. CECs degradation obeyed pseudo-first order kinetics (ln(C_t_/C_0_) = −*k*t, min^−1^).

### 2.7. Toxicity Assessment

The eco-toxicity of samples toward *Aliivibrio fischeri* was assessed following the standard ISO 11348-3 method and using the commercial kit BioFix^®^ Lumi-10 (Macherey-Nagel GmbH & Co. KG, Duren, Germany) by monitoring changes in bacteria bioluminescence after 30 min of contact time [21], and expressed as bioluminescence inhibition percentage (BI %). Samples were previously filtered (0.22 μm); pH value and salinity were adjusted to ≈7 and 2% (*w*/*v*), respectively. All water samples were analyzed with the PMS contents of 1 mmol/L; therefore, to avoid instant toxic effects of PMS, they were diluted 1:10 (*v*/*v*); this dilution factor is below the range of 1:80 to 1:100 *v/v* usually considered in real UWW coastal discharge [22].

Phytotoxicity tests were performed following standard procedures for root lengths assessment in *Sorghum saccharatum* (Sorgho), *Lepidium sativum* (garden cress), and *Sinapis alba* (mustard), using seeds provided by a commercial kit (Phytotoxkit liquid samples, Microbiotests Inc., Gent, Belgium) [21,23]. A water solution of Zn^2+^ (100 mg/L) and fresh-collected UWW were used as positive and negative toxicity control, respectively. Tests were performed by adding 8 mL of a water sample on Petri dishes (140 mm) lined with two sterilized filter paper layers (0.39 mm thick). A total of 10 seeds (only 5 for *S. saccharatum*) were equally distributed in each Petri dish and incubated for 72 h in darkness at 25 ± 1 °C. Then, the radicle lengths were measured to calculate the relative growth index (RGI) as a ratio of radicle length of samples/negative control. Results can be classified as: (i) inhibition (toxic effect) for 0 < RGI < 0.8; (ii) no significant effect for 0.8 ≤ RGI ≤ 1.2; and (iii) stimulation (benefit): RGI > 1.2 [24].

## 3. Results and Discussion

### 3.1. Simultaneous Inactivation of Bacteria and Degradation of CECs in SUWW

**Figure 1:**
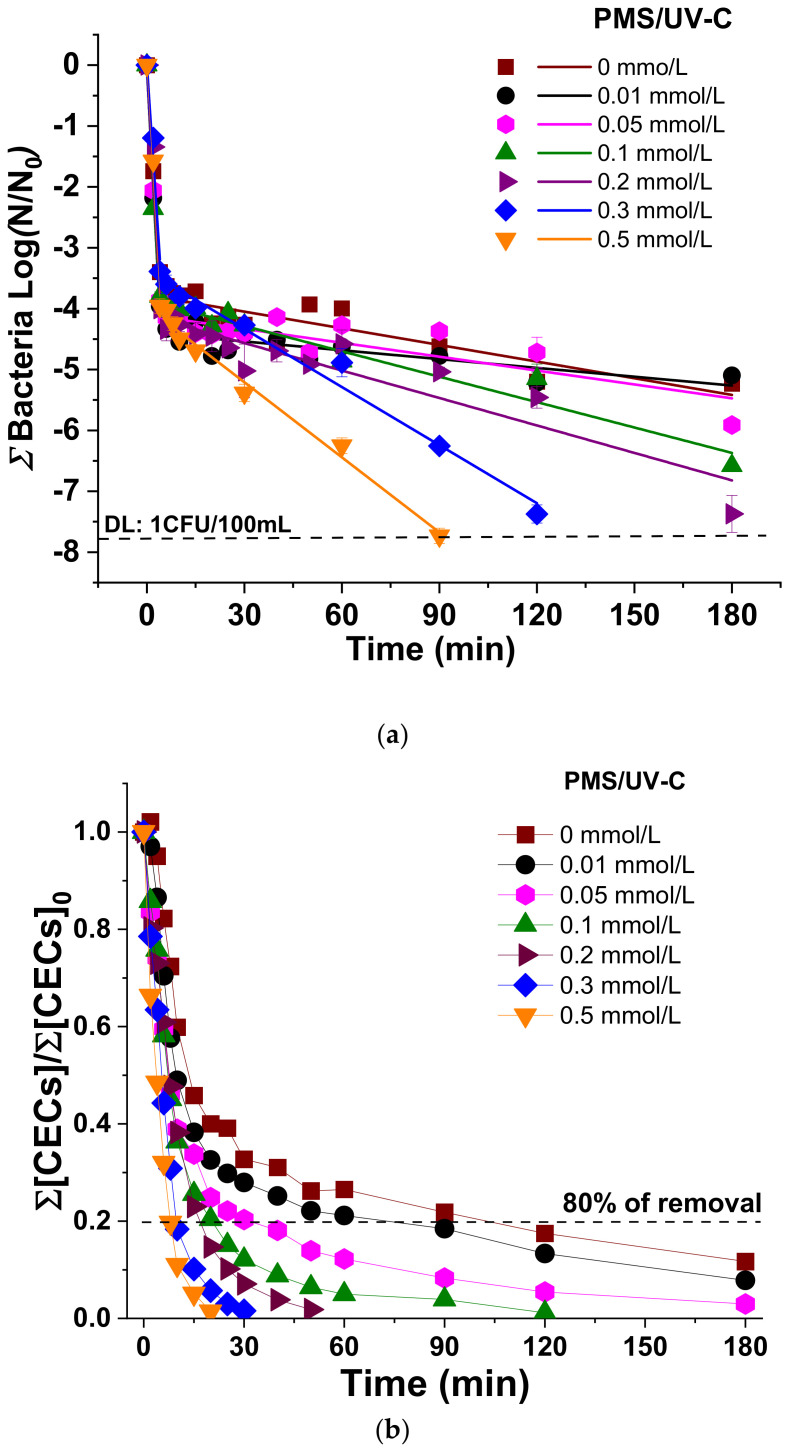
(**a**) Linear fitting of total bacteria inactivation; (**b**) Degradation of total CECs in SUWW under UV-C irradiation in the presence of increasing concentration of PMS (0–0.5 mmol/L).

Figure 1 shows the simultaneous inactivation of the sum of all bacteria (Figure 1a) and the degradation of total CECs (Figure 1b) in SUWW by UV-C radiation and PMS/UV-C with concentrations of PMS ranging from 0.01 to 0.5 mmol/L. Tables S3 and S4 report the corresponding inactivation and degradation kinetic constants, respectively, and Figure S2 shows individual profiles for each target.

The bacterial inactivation profiles observed in all cases obey to a double log-linear kinetic profile explained by the action of the UV-C radiation (a fast *k*_1_) and the experimental setup (slower *k*_2_) (Table S3, Figure S2), which is similar to previous studies using the same UV-C pilot plant [16]. *k*_1_ is attributed to the well-known efficiency of UV-C wavelengths to inactivate microorganisms in water. As it is observed in our results, no significant differences on the three individual bacterial profile were obtained, attaining 3.4 LRV (Logarithm Reduction Value) in the first 4 min of treatment time. During this first stage, the inactivation mechanism is attributed to the direct photoabsorption of nucleic acids (maximum absorption at 260 nm), with consequent bacterial DNA damages. Upon irradiation, adjacent pyrimidine nucleobases can dimerize, inducing the formation of cyclobutane pyrimidine dimers (CPDs), and the formation of pyrimidine (6-4) pyrimidine dimers [9]. On the other hand, a second kinetic rate (*k*_2_) involving the remaining microbial concentration (up to 4 log, considering a detection limit of 1 CFU/100 mL) clearly showed a process limitation mainly attributed to the flow mode used in this study [16], with very low kinetic rates and not reaching the DL for any of the bacteria investigated. No significant differences between the inactivation profiles of the three bacteria were observed according to *k*_2_ (Table S3, Figure S2). This result indicates that the reduced efficiency of UV-C process alone to reach the DL could be attributed to the photo-limitation by the configuration of the system (re-circulation flow-mode and low ratio total/illuminated water volume). Consequently, a post-treatment bacterial regrowth analysis after 48 h revealed the presence of all bacteria.

Regarding the simultaneous CECs abatement by UV-C alone (Figure 1b), the results showed that 80% removal of total CECs was attained after up 120 min of treatment (2.9 kJ/L), but significant differences of the individual CEC susceptibility and removal was observed (Figure S2): DCF > SMX > TMP. This result is explained by the well-known influence of the molar absorption coefficient of each CEC and the quantum yield at the wavelength of lamp emission (ε254 and Φ254, respectively) to favor the breakdown of the compounds under radiation, concluding therefore that CECs removal by UV-C alone is highly influenced by the physicochemical structure of each compound. According to the literature, SMX has higher ε254 (16500 and 6100 1/M·cm for SMX and DCF, respectively) but 8-fold lower Φ254 than DCF (0.058 and 0.222 mol/Es for SMX and DCF, respectively), and for this reason, it exhibits a lower photolysis efficiency. TMP values of ε254 and Φ254 are low (2900 1/M·cm and 0.006 mol/Es), which may explain the lower removal rate observed with only UV-C radiation [16].

The disinfection and decontamination results obtained by UV-C alone indicate that even though this process is highly efficient for water disinfection, important limitations still remain, such as the residual microbial load and the regrowth after the treatment, as well as its limited CECs removal. Therefore, the combination of UV-C with a chemical oxidant to promote the generation of ROS to improve both CECs and bacterial abatement is necessary to safely obtain reclaimed water.

In this study, the employ of PMS as a promoter of radical’s generation under UV-C determined a significant enhancement in bacteria inactivation and CECs degradation, and the testing of increased concentration from 0.01 to 0.5 mmol/L showed that the higher the PMS concentration, the faster the removal kinetics rates (Figure 1a, Tables S3 and S4).

The detection limit of the sum of all bacteria (8 LRV,DL: 1 CFU/100 mL) was achieved for PMS concentrations higher than 0.2 mmol/L, which is also the minimum concentration necessary for no observing bacterial regrowth after 24 and 48 h of post-treatment. At these PMS concentrations (0.2–0.5 mmol/L), 4.0±0.2 LRV of total bacteria was achieved after 4 min with a *k_1_* of (0.90 ± 0.20) min^−1^, obtaining slightly enhanced results compared with the UV-C process alone (3.4 LRV and a *k*_1_ of 0.85 ± 0.20 min^−1^). However, the significant positive effect of the use of PMS is clearly observed in the second phase of the process (*k*_2_), DL reached and no-regrowth (Figure 1a, Figure S2 and Table S3). The individual bacterial behavior (Figure S2) revealed the following order of bacterial inactivation: *E. coli* (Gram negative) ≈ *P. aeruginosa* (Gram negative) ≥ *E. faecalis* (Gram positive), which is similar to other studies reported in the literature and mainly attributed to the thicker cell wall of the Gram positive bacteria [16].

Regarding CECs removal, a significant enhancing of the removal rate of the total load when increasing the PMS concentration was obtained (Figure 1b). Focusing on each of the CECs (Figure S2), it is observed that this overall enhancement is due to the susceptibility of TMP to the PMS/UV-C process, while in the case of SMX and DCF, no significant differences were obtained in the presence and absence of PMS.

Considering simultaneous water disinfection and decontamination, the best performance was reached at 0.5 mmol/L of PMS, obtaining 8 LRV for total bacteria after 90 min of treatment (2.1 kJ/L) and 80% of CECs removal after 8 min (0.02 kJ/L). The enhancement water purification by the PMS/UV-C process is attributed to the generation of SO_4_^•−^ and ^•^OH radicals by PMS photolysis [25]. These ROS initiate oxidative stress by attacking firstly the cell membrane and altering its permeability, although they can also permeate easily through the cell-wall membrane and further react with cellular components (enzymes and genetic materials), inhibiting normal metabolism and thus leading to cells’ inactivation [26]. Regarding CEC, the degradation is mainly initiated by SO_4_^•−^ and ^•^OH attack with second-order kinetic constants in the range of 10^9^ M^−1^ s^−1^ [27]. However, the results demonstrated that DCF and SMX are not chemically limited, and increasing PMS concentration did not enhance significantly their degradation, which is mainly due to an important effect of only UV-C radiation on the removal of these targets (Figure S2b,d). This could be explained considering ε, Φ, and the concentration of targets and oxidant. Herrmann et al. (2007) reported values of 0.012 and 19.1 L/mol cm for ε and Φ of PMS, respectively [28]. Despite the PMS concentration, being 10^4^ times higher than CECs, DCF and SMX were able to absorb directly UV-C absorption of light due to the higher values of ε and Φ [16] and photolysis is the main cause of degradation. For this reason, an increase in oxidant concentration did not involve a significant enhancement in their degradation. On the other hand, TMP degradation was mediated by SO_4_^•−^ and ^•^OH, in which PMS is more efficient than direct UV-C light absorption.

### 3.2. Simultaneous Inactivation of Bacteria and Degradation of CECs in UWW

**Figure 2:**
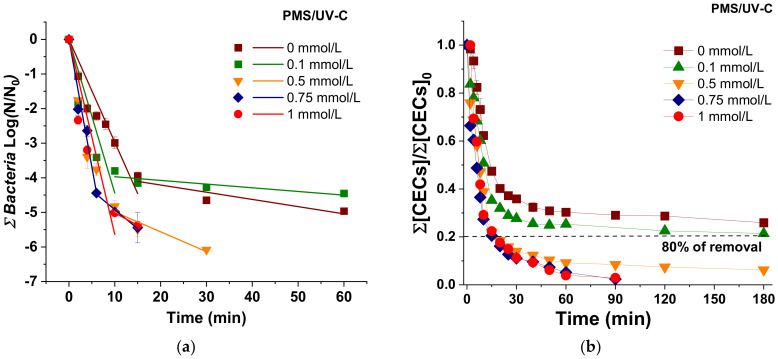
(**a**) Linear fitting of sum of bacteria inactivation; (**b**) Degradation of total CECs in UWW under UV-C irradiation in the presence of increasing concentration of PMS (0–1 mmol/L).

The simultaneous inactivation of the total bacteria (including *E. coli*, *Enterococcus* spp., and *Pseudomonas* spp.) (Figure 2a) and the degradation of total CECs (Figure 2b) under UV-C radiation and PMS/UV-C at oxidant concentrations ranging from 0 to 1 mmol/L has been investigated in UWW. Kinetic rate analysis and individual profiles of each target are shown in Tables S5 and S6 and Figure S3, respectively. The results obtained in this water matrix followed the same trend than the observed in SUWW; i.e., a limited process performance of UV-C alone was obtained. DL for bacterial inactivation was not reached, and low CECs removal kinetic rates were obtained; meanwhile, the addition of increasing PMS concentrations determined a great enhancement on the simultaneous CECs degradation and bacterial inactivation, detecting no regrowth of microbial targets only with concentrations higher than 0.5 mmol/L. In addition, concentrations higher than 0.5 mmol/L were investigated to determine the best process performance considering the higher physicochemical and microbiological complexity of the UWW water matrix. Nevertheless, results showed no significant differences between 0.5, 0.75, and 1 mmol/L of PMS for total bacteria inactivation and CECs removal. Beyond the optimum, an enhancement was not observed due to the possible reactions between oxidant and radicals and self-recombination of active species that could exceed the ones with biological and chemical targets, as it is reported elsewhere [25].

However, focusing on each individual target, this trend is also observed except for TMP, where higher PMS concentration than 0.5 mmol/L was necessary to achieve 80% of removal (Figure S3f). Therefore, these results clearly state that depending on the desired final results or quality requirements established by reclamation guidelines, the optimization of the oxidative agent concentration could vary, which also directly affects the treatment cost.

Considering the simultaneous UWW disinfection and decontamination (Figure 2), the best PMS concentration was also obtained with 0.5 mmol/L. At this condition, 4.7 LRV (concentration < 10 CFU/100 mL) was achieved after 10 min of treatment (0.01 kJ/L) for all bacteria, and 80% of total CECs was removed after 15 min (0.04 kJ/L). For comparison, in SUWW, 80% of CECs was removed in half of the time (after 8 min and 0.02 kJ/L), while 4.5 LRV were obtained in a similar treatment time (10 min), which could be attributed to the higher load of microbial targets in SUWW in comparison with UWW.

It is well recognized that the water matrix significantly affects disinfection and decontamination efficiency [29]. The main natural constituents of different water matrices include natural organic matter (NOM) and inorganic species, such as Cl^−^, CO_3_^2−^/HCO_3_^−^, SO_4_^2−^, and NO_3_^−^. These components are ubiquitous in UWW at different concentrations and they can be involved in side reactions, promoting or inhibiting the process. Lower degradation rates could be related to PMS consumption by water constituents. In fact, PMS is able to directly oxidize Cl^−^ into less reactive chlorine species (such as Cl_2_ and ClO^−^), and it is able to react with organic matter, as it is has been described in the literature [25,30].

Another inhibiting effect could be correlated to light attenuation, which is induced by the high light absorption of NOM at 254 nm. This filtering effect can be observed in Figure S1, showing different irradiance (W/m^2^) profiles under UV irradiation in DW, SUWW, and UWW, with lower values measured in the more complex matrix. Lower oxidant availability and light attenuation induce also a decrease of the active species (SO_4_^•−^ and ^•^OH) generated in solution. A further reduction is due to the scavenging effect, with a side reaction between inorganic species (mainly Cl^−^ and CO_3_^2−^/HCO_3_^−^) and NOM with active radicals (SO_4_^•−^ and ^•^OH), converting them into less reactive species (Cl^•^/Cl_2_^•−^ and CO_3_^•−^). Among the inorganic species, SO_4_^2−^ is not considered a robust radical scavenger due to its slower reaction with active radicals compared to other anions. On the other hand, NO_3_^−^ and NOM, under certain conditions, could act as photosensitizers, absorbing light, generating ROS in solution, and increasing degradation rates [25]. Wacławek et al. (2017) reported second-order reaction rate constants of SO_4_^•−^ and ^•^OH with common ions and NOM (humic acids). They report that k^•^_OH,inorganic ions/NOM_ are 10 times higher than k_SO4•−,inorganic ions/NOM_, and for this reason, SO_4_^•−^ exhibits high selectivity in a complex water matrix [12].

UV-C driven SR-AOPs have been studied for the degradation of different contaminants and for the inactivation of several pathogens, but only few studies involve the use of complex matrices [14,15,31,32,33]. Mahdi-Ahmed et al. (2014) reported Ciprofloxacin (CIP) oxidation by UV-based AOPs, comparing the performances of three oxidants, PMS, PS, and H_2_O_2_ in DW and UWW. UV-based AOPs significantly increased the removal efficiency in DW with all the oxidants being the order of efficiency UV/PS > UV/PMS > UV/H_2_O_2_ > UV. However, in a complex water matrix, the most efficient process was UV/PMS followed by UV/PS and UV/H_2_O_2_, because SR-AOPs, involving PMS and PS, were less affected by the presence of NOM and inorganic species, which was due to the limited scavenger effect toward the higher selective SO_4_^•−^. Moreover, they correlated the higher PMS reactivity to the presence of HCO_3_^−^, which is capable of activating PMS. CIP (at an initial concentration of 50 µmol/L = 17 mg/L) was fully degraded in UWW at pH 7 in 60 min in the presence of 1 mmol/L of PMS under UV-C irradiation [31]. Rodriguez-Chueca et al. (2018) investigated among other tertiary treatments (coagulation/flocculation, filtration, and UV-C radiation with different oxidants), the PMS/UV-C process to remove 25 CECs (including antibiotics, pesticides, flame retardants, corrosion inhibitors, and synthetic fragrances) detected at trace level (1–4 µg/L). They tested different oxidant concentrations (0.05, 0.2, and 0.5 mmol/L) and contact times (4–18 s), reporting average removal rates of CECs of 55%, 48%, and 10% for H_2_O_2_, PMS, and PS, respectively, compared to 13% with only UV-C radiation [14].

Higher treatment efficiencies than those reported in the literature were obtained in our study due to the use of a batch mode system that allowed higher contact times compared to the treatment performed in a continuous flow mode, which is commonly employed in real scenarios. However, it is important to consider that the effectiveness of a UV system depends on different parameters, such as the intensity of radiation, the contact time, the reactor configuration, and the composition of the water matrix. Systems in a continuous flow-mode are actually commonly used for disinfection purposes in drinking water treatment plants for the treatment of high-quality water characterized by low turbidity and asmall content of suspended solids, and NOM. In a complex matrix, it is necessary to take into account that disinfection and decontamination could be ineffective due to low contact times, photons scattering, and absorption effects by constituents of the water matrix, leading to a low CECs degradation efficiency and high probability of microbial regrowth.

Rodriguez-Chueca et al. (2019), in a latter work, also pointed out that the process efficiency depends on the target compound and the experimental conditions (contact time and oxidant concentration) by studying the degradation of atenonol, bisphenol A, caffeine, carbamazepine, diclofenac, ibuprofen, sulfamethoxazole, and their transformation products with low dosage of oxidant (0.5 mmol/L), obtaining good removal efficiencies only for some of them [34].

### 3.3. ARB and ARGs Removal in UWW

**Figure 3:**
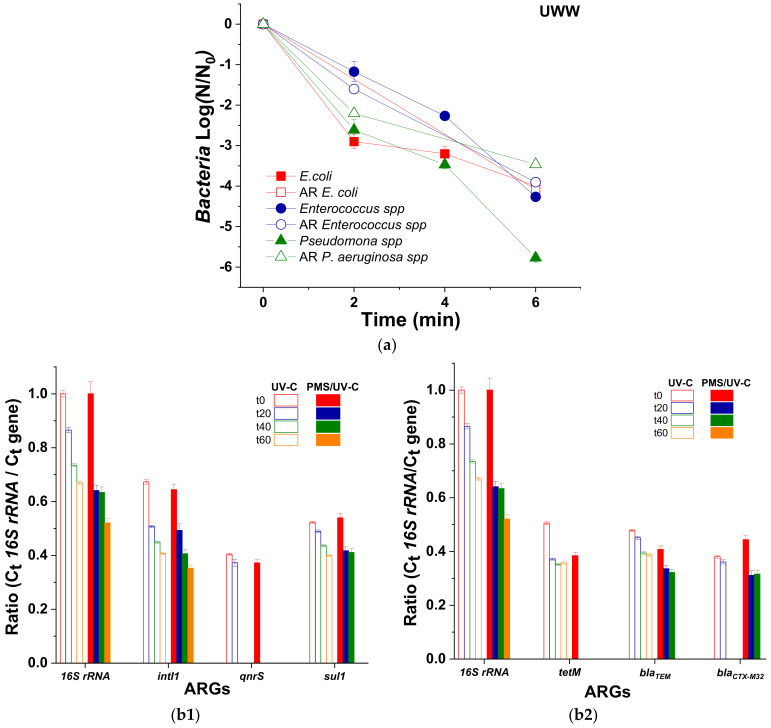
(**a**) Inactivation of *E. coli*, *Enterococcus* spp., and *Pseudomonas* spp. and their Ampicillin (AMP), Ciprofloxacin (CPX) and Trimethoprim (TMP) antibiotic-resistant counterpart and (**b1**,**b2**) relative abundance of each ARG (with respect to *16S rRNA*) as a function of time under UV-C irradiation in the absence and in the presence of PMS (1 mmol/L).

Currently, the high concern of the scientific community about ARB and ARGs spreading has promoted the research of water treatment methods able to efficiently remove these biological targets from UWW effluents. In this study, the inactivation of ARB and the degradation of ARGs after UV-C treatment with PMS has been therefore also assessed. Figure 3a shows the inactivation of *E. coli*, AR-*E. coli*, *Enterococcus* spp., AR-*Enterococcus* spp., *Pseudomonas* spp., and AR-*Pseudomonas* spp. in UWW by PMS (1 mmol/L)/UV-C radiation. The high oxidant concentration used in the present work was selected considering previous studies [32,35,36]. Arslan-Alanton et al. (2021) could not detect quantifiable DNA for the genes *16S rRNA*, *aphA* and *tetA* as a result of the application of PMS/UV-C treatment at a concentration of 2 mmol/L of oxidant in tertiary treated UWW [32]. In addition, a review by Michael-Kordatou et al. (2018) determined that the necessary concentration of oxidant for the removal of ARGs in wastewater effluents using different advanced chemical oxidation processes is higher than the corresponding concentration to inactivate ARB [35].

Our results showed that all the wild and AR bacteria determined in this study reached the DL (1 CFU/100 mL) in 6 min (Q_UV_ of 0.01 kJ/L). At this time and under such operational conditions, 3 LRV of *E. coli*, AR-*E. coli*, *Enterococcus* spp., and AR-*Enterococcus* spp. were attained; while 6 LRV of *Pseudomonas* spp. and 3 LRV AR-*Pseudomonas* spp. (due to its lower initial concentration) were obtained. These results agree with a few works reporting ARB inactivation in UWW by PMS/UV-C, where 5.3 LRV of AR-*Pseudomonas* sp. HLS-6 was obtained after 10 min of treatment (60 mJ/cm^2^) with 1 mg/L of PMS [37]. In addition, no significant differences in the resilience between ARB and the wild-type bacteria were obtained (Figure 3a), which agree with other studies in the literature for different oxidative processes. As already demonstrated by Fiorentino et al. (2019), the same inactivation kinetics were obtained for wild-type bacteria than for ARB in UWW after the application of a solar photo-Fenton process using raceway pond reactors at neutral pH, pointing out that the inactivation of ARB is not the main issue in the treatment of wastewater, but it is the removal of ARGs [38].

The use of antibiotics in humans and animals over the years has produced ARB and encoding ARGs. Nowadays, different types of ARGs exist in the environment, such as genes conferring resistance to quinolone, tetracycline, sulfonamide, β-lactamase, cephalosporine, etc. The resistance bacteria to these antibiotics emerge from environmental exposure, as tetracycline or β-lactamase, and also from their widespread use to treat bacterial infections in both human and animal clinics, such as quinolones and sulfonamides [39,40,41]. Therefore, the occurrence ratio of different ARGs related with these antibiotics was initially determined in the secondary effluents of UWW from El Bobar (Almeria, Spain). It was calculated by means of their relative abundance by analyzing the *16S rRNA* gene, which represents the microbiological abundance in UWW. The following rank was obtained: *intI1* (0.654 ± 0.021) > *sul1* (0.530 ± 0.014) > *bla_TEM_* (0.443 ± 0.042) > *tetM* (0.440 ± 0.049) > *bla_CTX-M32_* (0.408 ± 0.091) > > *qnrS* (0.384 ± 0.021) (Data from Table S7).

The gene of class 1 integron integrase, *intI1*, is considered an indicator of horizontal gene transfer. For this reason, it is very important to attain its degradation, because *intI1* encodes a protein involved in the integration of DNA into the cell [42,43].

Throughout the PMS/UV-C radiation treatment at 1 mmol/L of oxidant, the ARGs showed different grades of degradation (Figure 3b). The quantification limit (QL) was achieved after 20 min of treatment (0.3 kJ/L) for *tetM* and *qnrS* genes and after 60 min (1.4 kJ/L) for *sul1*, *bla_TEM_*, and *bla_CTX-M32_*, while 50% of degradation was observed for the *16S rRNA* and *intI1* genes after 60 min of treatment time. However, UV-C alone, showed different grades of degradation. The QL is only reached for the *qnrS* and *bla_CTX-M32_* genes after 40 min (0.9 kJ/L), while for the other genes, degradation percentages of 38.8%, 33%, 29.4%, 23% and 18.8% were obtained after 60 min of treatment for *intI1*, *16srRNA*, *tetM*, *sul1*, and *bla_TEM_*, respectively. Comparing the results of both UV-C and PMS/UV-C, it can be determined that the highest degradation of ARGs is obtained when PMS oxidant is present.

Very few studies in the literature report on the removal rate of ARGs in UWW by UV-C and its combination with PMS. Rodríguez-Chueca et al. (2019) reported ARGs (*bla_TEM_*, *sul1*, and *qnrS*) removal in UWW by different oxidation processes (UV-C alone, UV-C/H_2_O_2_ and UV-C/PMS). They concluded, from the results obtained after 4 s of contact time (0.41 log, 0.21 log, and 0.22 log), that the most effective treatment for the removal of ARGs is UV-C radiation alone, which is followed by UV-C/PMS (at 0.5 mmol/L). This effect was attributed to the UV photons scavenger by oxidants in detriment to DNA photoabsorption, reducing the direct damage to the genes. In addition, the LOQ of the investigated genes was not reached probably due to the very low contact time (4 s of UV-C irradiance) [33], which were much lower than those covered in the present study (60 min of treatment time, corresponding to 300 s of UV-C irradiance).

Hu et al. (2019) reported reduction rates of 2.9 log and 3.4 log for *sul1* and *intI1* genes, respectively, after the treatment with UV-C (180 mJ/cm^2^) and 20 mg/L of PMS during 30 min in 50 mL of phosphate buffer saline solution. They also compared different treatments (UV-C, PMS, and UV-C/PMS) for both genes obtaining a lower ARGs reduction rate of 1.2 log and 0.8 log (*sul1* and *intI1*, respectively) after 30 min of UV-C treatment alone. Hence, they concluded that SO_4_^•−^ radicals were responsible for the reduction of genes by direct attack to DNA [37].

Likewise, Arslon-Alanton et al. (2021) reported the LOQ for genes *16S rRNA*, *aphA,* and *tetA* after the treatment of 500 mL of tertiary UWW with UV-C (0.45 W/m^2^) and the oxidant PMS (2 mmol/L) at a contact time of 80 min. The authors also compared the application of the treatment UV-C alone, obtaining only a removal rate of 3.27 ± 0.07 log for *16S rRNA* and 2.99 ± 0.44 log for *aphA*, concluding that the addition of strong oxidation agents such as PMS enhances the removal of ARGs in tertiary UWW [32].

The results of the present study on the removal of ARGs determine that UV-C treatment with the oxidant PMS is more efficient than UV-C alone.

It is well known that the UV-C radiation induces a germicidal effect by producing the formation of cyclobutane pyrimidine dimers (CPDs) in the DNA. However, according to the literature consulted, it is not already experimentally demonstrated that the SO_4_^•−^ produced during the treatment with UV-C causes damage to ARGs [9].

The results obtained for ARB inactivation and ARGs removal after PMS/UV-C treatment demonstrate its promising efficiency; an exposure time of 60 min was necessary to achieve 50% removal of the *16S rRNA* and *intI1* genes and the quantification limit for the *sul1*, *bla_TEM_*, *bla_CTX-M32_*, *qnrS* and *tetM* genes. The inactivation of ARB alone does not guarantee DNA damage, which may be present and undamaged, being able to contribute to antibiotic resistance spread through different transmission mechanisms. Therefore, it is important to consider both ARB and ARGs as unique targets in future disinfection strategies.

### 3.4. Toxicity Assessment

**Figure 4:**
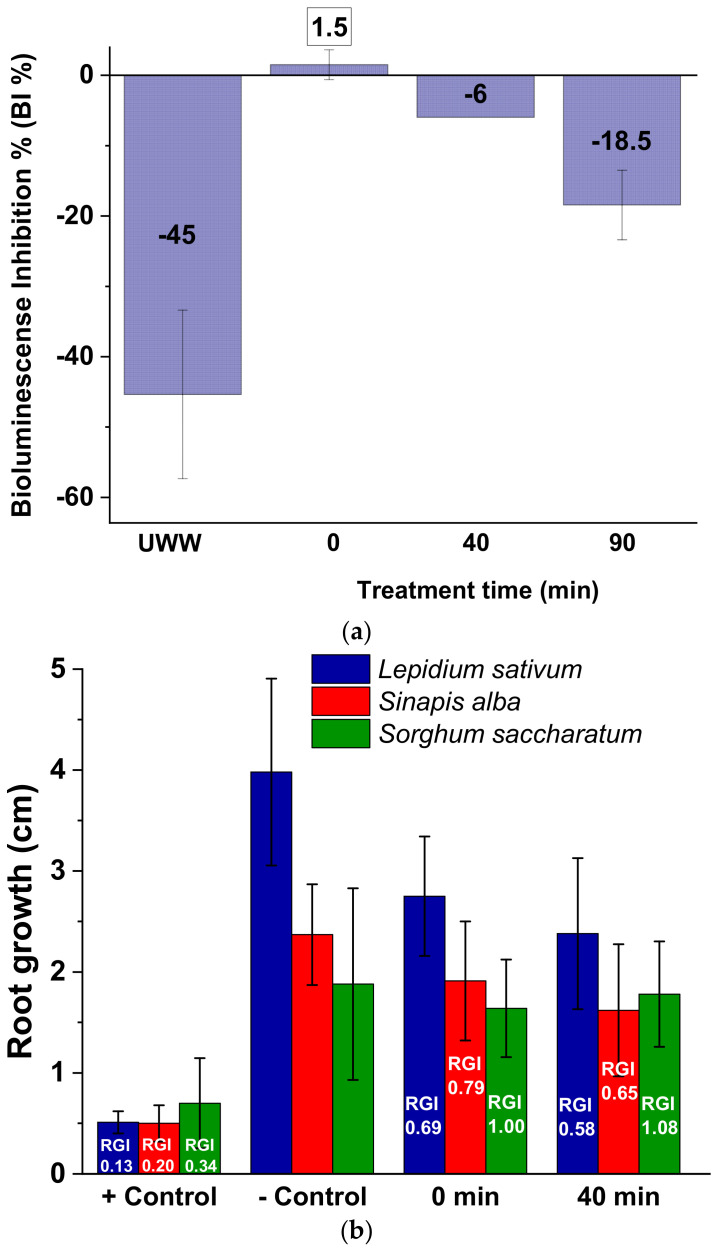
(**a**) *A. fischeri* bioluminescence inhibition (BI) (negative values state for stimulation effect instead of inhibition) and (**b**) phytotoxicity test in Zn^2+^ solution (positive (+) control), UWW (negative (−) control), and UWW samples treated by PMS/UV-C (1 mmol/L).

Figure 4a shows the bioluminescence inhibition (BI) results toward *A. fischeri*. It is observed that the luminescence emitted by the marine bacterium was not significantly affected, as the BI % values were −44.5 ± 12, 1.5 ± 2.1, −6 ± 0, and −18.5 ± 4.9 for the UWW matrix, UWW with PMS addition (Time 0), and treated UWW (40 and 90 min of treatment time), respectively. An eco-toxicity result of <20% indicates that the discharge of UWW treated by PMS/UV-C does not pose a harmful effect to the receiving aquatic environment (non-acute toxicity) [44]. Nevertheless, it is also important to note that the mere effect of PMS addition led to a significant bioluminescence decrease with respect to the UWW matrix (46 BI % increase) followed by a toxicity reduction (BI % decrease) at the end of the treatment time. A similar effect has been previously reported by Deng et al. (2017) in river water with 1 mmol/L of PMS. They found 65 BI % at the same PMS concentration and a decrease in the toxic effect along the treatment time [45]. Results obtained in the present study also agree with the toxicity of PMS to microorganisms reported by the European Chemicals Agency (ECHA), where an EC_50_ value of 179 mg/L was reported for a growth inhibition test of the Gram negative bacterium *P. putida* [46].

Figure 4b shows the phytotoxicity results for *L. sativum* and *S. alba*, indicating a growth inhibition with respect to the negative control (UWW) for both untreated (0 min) and treated UWW (40 min): root growth length ca. 30 and 20% lower after PMS addition (RGI values) and an additional reduction of ca. 10% after UV exposure, respectively. No significant effects in *S. saccharatum* tests for 0 min samples or 40 min simples were observed.

Regarding phytotoxicity assessment, it is important to note that the PMS salt suffers an abiotic decomposition, being rapidly decomposed into products of no concern and ubiquitously present in the environment (potassium, hydrogen sulfate, hydrogen peroxide, and oxygen) upon contact with soils (DT_50_ < 11 min and no detection after 1 h) [46]. Therefore, the phytotoxicity results obtained should be taken into account as another trophic level of environmental toxicity assessment or as representative results but only for agriculture–aquaculture systems. According to the results obtained after PMS/UV-C treatment (Figure 4b, 40 min), the treated UWW generated had a slightly toxic effect (RGI < 0.8) toward *L. sativum* and *S. alba*, whereas a non-toxic effect (0.8 ≤ RGI ≤ 1.2) was observed for *S. saccharatum*. The weakest response by *Sorghum* and the high sensitivity of the other two plant species observed has also been previously reported indicating the suitability of *L. sativum* and *S. alba* as indicators for the assessment of the biological effect on plants and the lack of suitability of very resistant species such as *Sorghum* [47,48,49].

Another inherent issue associated to PMS addition may be the release of sulfate and potassium ions. Under tested experimental conditions and after PMS (1 mmol/L)/UV-C treatment, increases in the UWW concentrations of potassium and sulfate ions of ca. 85 and 165 mg/L were observed, respectively. Regarding a potential UWW discharge in coastal or surface waters, limits neither for potassium nor for sulfate are established. The final concentration after the treatment is lower than the level to impart undesirable taste in drinking water (250–500 mg/L) and to cause illnesses such as diarrhea (>1000 mg/L) [50].

On the opposite side, when the intended end use of the treated WW is agricultural reuse, the presence of a higher potassium concentration than the usual range in irrigation water (0–2 mg/L) and an additional sulfate concentration represent an added benefit to the farmer through the saving of essential fertilizers [51].

### 3.5. Cost Analysis

Table 1 shows the estimation of the annual treatment costs (ATC) associated with the implementation of the UV-C and PMS/UV-C processes as tertiary UWW treatments (after the biological step) in an already working UWWTP, taking into account a scaling-up from the results and the UV-C pilot plant used in the study (total volume 80 L, flow rate 36 L/min, irradiance 0.2 W/m^2^, and 6 L of illuminated volume). The calculations were done for the treatment of ca. 1000 m^3^/day in a continuous flow UV-C reactor, i.e., considering only a single irradiation step of 2 min total treatment time, 10 s UV-C contact time, and 12 mJ/cm^2^ UV-dose.

The investment, operational, and maintenance costs were estimated according to the United States Environmental Protection Agency (USEPA) report considering the UV-C dose of 12 mJ/cm^2^ (6 lamps required), a medium flow rate (USEPA category 3) of ca. 1000 m^3^ (0.27 MGD, million gallon day), and a capital recovery factor (CRF) of 0.117 (cost amortized at 10% for 20 years) [52].

The ATC value estimated for UWW treatment by UV-C alone was 8514 €, which lead to a treatment cost of 0.02 €/m^3^. Whereas the costs for PMS/UV-C treatment increased to 0.33, 0.48, and 0.63 €/m^3^ for 0.5, 0.75, and 1 mmol/L of PMS, respectively. This increment is mainly consequence of the reagent cost (2 €/kg or 0.61 €/mol of commercial PMS salt), which represents > 95% of the total treatment cost. Costs associated to an intermediate tank (to dissolve the PMS salt) and a dispenser pump were not considered due to their costs being negligible with respect to reagent costs. The estimated treatment costs are in agreement with the previous one reported in the literature for UWW: 0.012 and 0.585 €/m^3^ for UV-C and UV-C/PMS (0.5 mmol/L) [15].

The figure of merit Electrical Energy per Order (EEO) (Table 2) was also calculated (Equation (2), [55]) for both disinfection and CECs removal under the experimental conditions of the present work, as another usual descriptor to compare different treatments.

(2) $$EEO=\frac{P\left(kW\right)}{F\;\left(\frac{m^{3}}{h}\right)\times \log\;\left(\frac{C_{i}}{C_{f}}\right)}$$

where *P* is the rated power (0.3 kW, lamp (0.23 kW) + pump (0.07 kW)), *t* is the treatment time (2 min for flow conditions and the treatment time needed to achieve the Swiss guideline treatment goal (80% of removal) for batch or recirculation mode) [18], *V* is the treated volume (80 L), and *C_i_* and *C_f_* are the initial and final concentration of the target for both UV-C and PMS/UV-C (0.5 mmol/L) (Table S8).

The efficiency data used to compare both treatments in terms of EEO are shown in Table S8. Lower energy requirements (kWh/m^3^) for both treatments goals (disinfection and CECs removal) and operational modes (flow and batch) were obtained for the PMS/UV-C process. For the continuous flow mode, significant lower energy requirements (≥ 3 and 13 times lower for disinfection and CECs removal, respectively) and significant higher treatment efficiencies (increases of > 1 *E. coli* LRV, > 25% of CECs removal) were observed for the PMS/UV-C process with respect to the conventional UV-C process alone. Moreover, if the treatment goals are taking into account (quality regulations/guidelines), which are linked with higher UV-C doses (higher treatment times through batch mode operation), the energy requirements are not significantly higher with respect to the continuous flow mode for the PMS/UV-C process (0.08 to 0.20 kWh/m^3^ and 1.04 to 1.07 kWh/m^3^ for disinfection and CECs removal, respectively).

According to the treatment costs obtained, it is clear that based only on economic aspects, the combination of PMS with UV-C treatment is not economically competitive (mainly associated with reagents costs). Nevertheless, if the treatment efficiencies, the water quality guidelines requirements, and the energy demand associated (significant lower for PMS/UV-C) are considered (Table S8), the UV-C/PMS alternative becomes a promising process for the near future in both operation modes (continuous flow and batch).

## 4. Conclusions

The combination of PMS (as oxidant agent) with UV-C irradiation (a commonly tertiary disinfection treatment used in UWWTPs) has been demonstrated to successfully address wastewater reuse challenges regarding fecal bacterial load (*E. coli*, *Pseudomonas* spp., and *Enterococcus* spp.), ARB and ARGs removal, CECs degradation, toxicity, and costs.

The best PMS concentration was found to be 0.5 mmol/L, at which limits on bacterial concentration < 10 CFU/100 mL were achieved after 10 min of treatment (0.01 kJ/L) for all bacteria investigated, following the order of susceptibility according to their inactivation kinetic rate: *E. coli > Pseudomonas* spp. *> Enterococcus* spp. Regarding organic chemical pollutants, 80% of the total CECs analyzed (DCF > SMX > TMP) was removed after 15 min (0.04 kJ/L).

Higher oxidant load (1 mmol/L) effectively removed ARB and ARGs. Significant differences in the inactivation of wild and AR-bacteria were not observed, and DL of 1 CFU/100 mL was reached after 6 min of treatment (0.01 kJ/L) for all microbial targets. The quantification limit of the genes *sul1*, *bla_TEM_*, *bla_CTX-M32_*, *qnrS*, and *tetM* genes was reached within 60 min of treatment, while only 50% of the *16S rRNA* and *intI1* was removed, and higher efficiencies were obtained compared with UV-C alone. These results clearly highlight that ARB is not the challenge on UWW; rather, it is the removal of ARGs.

Toxicity studies were also performed to evaluate the oxidant harmful effect toward the receiving aquatic environment (considering effluent discharge) or for plants’ growth (considering effluent reuse in agriculture). A significant potential toxicity toward the aquatic ecosystem was not observed by evaluating *A. fischeri* bioluminescence inhibition, while the growth of *L. sativum* and *S. alba* was little affected. The transformation products generated during the treatment investigated in this study are also of concern, and they will be deeply analyzed in a further study.

From an economic point of view and comparing with UV-C alone, an increment in the cost for PMS/UV-C process, mainly due to the reagent price, is totally counterbalanced by a higher efficiency in the simultaneous removal of chemical and biological targets, avoiding bacterial regrowth and making wastewater reuse safer. Therefore, the PMS/UV-C process could be a suitable option to be implemented in UWWTPs.

## Figures and Tables

**Table 1:** Breakdown of the estimated UV-C and PMS/UV-C annual costs for UWW treatment in a continuous flow reactor.

Investment Costs	1301 €
Equipment cost (EC)	1085 €
Engineering and installation (20% of EC)	216 €
**Operational and Maintenance Costs**	**7213 €**
Replacement (lamps): 6 UV-C lamps (8760 h) × 820 €/lamp [53]	4920 €
Power: 4468 kWh (lamps) and 11,643 kWh (pumping) = 16,111 kWh × 0.12 €/m^3^ [54]	1933 €
Labor (36 h) (0.5 h per month per lamp for cleaning and repair) × 10 €/h	360 €
**UV-C Total Cost**	8514 €
	0.02 €/m^3^
**UV-C + 0.5 mmol/L PMS**	120,450 €
	0.33 €/m^3^
**UV-C + 0.75 mmol/L PMS**	176,414 €
	0.48 €/m^3^
**UV-C + 1 mmol/L PMS**	231,164 €
	0.63 €/m^3^

**Table 2:** Energy per order (EEO, kWh/m^3^) for UWW treatment (disinfection and CECs removal) in continuous flow (one path throughout lamp) and batch conditions by UV-C and PMS/UV-C (0.5 mmol/L) processes.

	EEO (kWh/m^3^)			
	*E. coli*		CECs	
	Continuous Flow (2 min)	Batch Mode (≤10 CFU/100 mL)	Continuous Flow (2 min)	Batch Mode (≥80% Removal ƩCECs)
UV-C	0.25	0.41	14.22	19.23
PMS/UV-C (0.5 mmol/L)	0.08	0.20	1.04	1.07

## Data Availability

Not applicable.

## Supplementary Materials

The following are available online, Table S1: Physicochemical and microbiological characterization of SUWW and UWW, Table S2: Conditions used in qPCR real-time assays, Table S3: Inactivation kinetic constants of *E. coli*, *E. faecalis*, and *P. aeruginosa* in SUWW, Table S4: Pseudo-first-order degradation kinetic constants of Trimethoprim (TMP), Sulfamethoxazole, and Diclofenac (DCF) in SUWW, Table S5: Inactivation kinetic constants of *E. coli*, Total Coliforms, *Enterococcus* spp., and *Pseudomonas* spp. in UWW, Table S6: Pseudo-first-order degradation kinetic constants of Trimethoprim (TMP), Sulfamethoxazole (SMX), and Diclofenac (DCF) in UWW, Table S7: ARGs means and standard deviation of the ratio detected in this study, Table S8: Treatment efficiencies achieved in continuous flow (after 2 min) and in batch mode (considering the treatment time to achieve *E. coli* ≤ 10 CFU/100 mL and 80% of removal of CECs) by UV-C and PMS/UV-C (0.5 mmol/L), Figure S1: UV-C lamp irradiance (W/m^2^) profiles registered in demineralized water (as baseline), SUWW and UWW, Figure S2: Inactivation profiles of *E. coli* (a), *E. faecalis* (c), and *P. aeruginosa* (e) and degradation profiles of DCF (b), SMX (d), and TMP (f) in the presence of increasing concentrations of PMS (0–0.5 mmol/L) and under UV-C irradiation in SUWW. Inserts: Enlargement of degradation curves, Figure S3: Inactivation profiles of *E. coli* (a), *Enterococcus* spp. (c), and *Pseudomonas* spp. (e) and degradation profiles of DCF (b), SMX (d), and TMP (f) in the presence of increasing concentrations of PMS (0–1 mmol/L) and under UV-C irradiation in UWW. Inserts: Enlargement of degradation curves, Figure S4: PMS concentration decrease in function of time (min) and accumulative UV energy Q_UV_ (kJ/L) under UV-C treatment at different PMS concentrations in SUWW (0.01–0.5 mmol/L) (a) and in UWW (0.1–1 mmol/L) (b).
