# Supplementary material for: UV-C Peroxymonosulfate Activation for Wastewater Regeneration: Simultaneous Inactivation of Pathogens and Degradation of Contaminants of Emerging Concern
Source: Molecules. 2021 Aug 12;26(16):4890. doi: 10.3390/molecules26164890 (PMC8398486; doi:10.3390/molecules26164890)
Supplement: Supplementary file 1 [file molecules-26-04890-s001.zip › molecules-1319552-supplementary.pdf]

Titled: UV-C Peroxymonosulfate Activation for Wastewater Regeneration: Simultaneous Inactivation of Pathogens and Degradation of Contaminants of Emerging Concern

Authors: Ilaria Berruti[1], Samira Nahim-Granados [1], María Jesús Abeledo-Lameiro[1], Isabel Oller[1,2] and María Inmaculada Polo-López[1,2,*]

Affiliations:

[1]CIEMAT-PSA, Carretera de Senés Km 4, 04200, Tabernas (Almería), Spain

[2]CIESOL, Joint Centre of the University of Almería-CIEMAT, 04120 Almería, Spain

***Correspondence**: mpolo@psa.es; Tel.: + 34 950 387900

Supplementary Materials

Table S1. Physico-chemical and microbiological characterization of SUWW and UWW.

Parameter	SUWW	UWW
DOC (mg/L)	11.1±2.5	19.6±2.8
BOD_5 (mgO_2/L)	-	16±5
COD ((mgO_2/L)	-	40±10
[HCO_3^-] (mg/L)	69.5±7.3	370.0±28.0
Turbidity (NTU)	2.5±0.2	9.1±9.3
pH	7.4±0.2	7.6±0.1
Conductivity (mS/cm)	1.5±0.1	2.4±0.1
Cl^- (mg/L)	356.7±4.4	436.7±80.0
NO_3^- (mg/L)	2.7±0.9	4.0±0.9
NO_2^- (mg/L)	-	6.0±±3.1
PO_4^{3-} (mg/L)	4.0±1.6	3.4±1.5
Br^- (mg/L)	-	2.8±0.1
SO_4^{2-} (mg/L)	99.1±0.8	101.5±22.7
Na^+ (mg/L)	255.2±1.6	252.0±13.4
NH_4^+ (mg/L)	7.6±1.0	39.3±7.7
K^+ (mg/L)	9.5±2.3	27.8±1.0
Ca^{2+} (mg/L)	18.6±3.4	88.5±11.5
Mg^{2+} (mg/L)	13.3±1.6	46.7±7.5
E. coli (CFU/mL)	-	$5.87 \cdot 10^3 \pm 1.02 \cdot 10^4$
Enterococcus spp (CFU/mL)	-	$1.41 \cdot 10^4 \pm 2.24 \cdot 10^4$
Pseudomonas spp (CFU/mL)	-	$3.60 \cdot 10^2 \pm 5.03 \cdot 10^2$
Total coliforms (CFU/mL)	-	$1.43 \cdot 10^4 \pm 2.14 \cdot 10^4$

Table S2. Conditions used in qPCR real-time assays.

Genes	Primer sequence	µM	bp	Conditions	Ref.
16s rRNA	FW: TCCTACGGGAGGCAGCAGT<br>RV: ATTACCGCGGCTGCTGG	0.50	195	50°C - 1 min (1X); 95°C - 3 sec, 60°C - 30 sec (40X); 95°C - 15 sec; 60°C - 1 min; 95°C -15 sec	Modified from [19]
intI1	FW: GATCGGTCGAATGCGTGT<br>RV: GCCTTGATGTTACCCGAGAG	0.50	196	50°C - 1 min (1X); 95°C - 3 sec, 60°C - 30 sec (40X); 95°C - 15 sec; 60°C - 1 min; 95°C -15 sec	Modified from [19]
qnrS	FW: GACGTGCTAACTTGCGTG<br>RV: TGGCATTGTTGGAAACTT	0.60	118	50°C - 1 min (1X); 95°C - 3 sec, 60°C - 30 sec (40X); 95°C - 15 sec; 60°C - 1 min; 95°C -15 sec	Modified from [19]
sul1	FW:CGCACCGGAAACATCGCTGCAC<br>RV: TGAAGTTCCGCCGCAAGGCTCG	0.30	162	50°C - 1 min (1X); 95°C - 3 sec, 60 °C - 30 sec (40X); 95°C - 15 sec; 60°C - 1 min; 95°C -15 sec	Modified from [19]
$bla_{CTX-M32}$	FW: CGTCACGCTGTTGTTAGGAA<br>RV: CGCTCATCAGCACGATAAAG	0.30	156	95°C - 10 min (1X); 95°C - 30 sec; 55°C - 1 min; 72°C - 1 min (40X); 72°C - 7 min; 60°C - 10 sec; 95°C - 10 sec (1X)	[20]
Tet(M)	FW: GCAATTCTACTGATTTCTGC<br>RV:CTGTTTGATTACAATTTCCGC	0.20	186	50°C - 1 min (1X); 95°C - 3 sec, 60°C - 30 sec (40X); 95°C - 15 sec; 60°C - 1 min; 95°C -15 sec	Modified from [19]
bla_{TEM}	FW:TTCCTGTTTTTGCTCACCCAG<br>RV:CTCAAGGATCTTACCGCTGTTG	0.20	113	50°C - 1 min (1X); 95°C - 3 sec, 60°C - 30 sec (40X); 95°C - 15 sec; 60°C - 1 min; 95°C -15 sec	Modified from [19]

Table S3. Inactivation kinetic constants of *E. coli*, *E. faecalis* and *P. aeruginosa* in SUWW.

[PMS] mmol/L	*E. coli* k (min^{-1})	*E. coli* R^2	*E. faecalis* k (min^{-1})	*E. faecalis* R^2	*P. aeruginosa* k (min^{-1})	*P. aeruginosa* R^2
**UV-C (Figure 1a)**						
0	0.960±0.162<br>0.016±0.002	0.919<br>0.866	0.747±0.084<br>0.010±0.002	0.963<br>0.753	1.010±0.194<br>0.008±0.002	0.847<br>0.648
0.01	1.130±0.210<br>0.003±0.002	0.884<br>0.139	0.921±0.036<br>0.004±0.002	0.995<br>0.214	1.170±0.206<br>0.009±0.002	0.913<br>0.556
0.05	1.110±0.193<br>0.008±0.002	0.915<br>0.375	0.911±0.041<br>0.007±0.002	0.994<br>0.691	1.140±0.210<br>0.009±0.001	0.896<br>0.833
0.1	1.120±0.191<br>0.027±0.004	0.918<br>0.820	0.911±0.017<br>0.011±0.002	0.999<br>0.797	1.170±0.241<br>0.021±0.003	0.881<br>0.817
0.2	1.140±0.154<br>0.022±0.005	0.947<br>0.724	0.864±0.134<br>0.013±0.002	0.931<br>0.774	1.200±0.199<br>0.023±0.003	0.922<br>0.776
0.3	1.000±0.189<br>0.067±0.007	0.900<br>0.916	0.752±0.110<br>0.031±0.003	0.938<br>0.903	1.030±0.237<br>0.047±0.017	0.856<br>0.742
0.5	1.240±0.219<br>0.062±0.005	0.911<br>0.944	0.845±0.098<br>0.040±0.005	0.960<br>0.871	1.270±0.226<br>0.057±0.011	0.910<br>0.790

DL= 1CFU/100mL *Shoulder length (SL)= 20 min ** SL= 50 min *** SL= 30 min

Table S4. Pseudo-first order degradation kinetic constants of Trimethoprim (TMP), Sulfamethoxazole and Diclofenac (DCF) in SUWW.

[PMS] mmol/L	TMP k (min^{-1})	TMP R^2	SMX k (min^{-1})	SMX R^2	DCF k (min^{-1})	DCF R^2
**UV-C (Figure 1b)**						
0	0.007±0.001	0.978	0.107±0.005	0.978	0.113±0.012	0.927
0.01	0.009±0.001	0.997	0.121±0.006	0.978	0.166±0.010	0.970
0.05	0.015±0.001	0.974	0.129±0.006	0.974	0.185±0.012	0.966
0.1	0.028±0.001	0.997	0.136±0.005	0.986	0.195±0.012	0.974
0.2	0.059±0.001	0.996	0.147±0.008	0.975	0.204±0.021	0.930
0.3	0.096±0.002	0.995	0.184±0.005	0.995	0.229±0.036	0.869
0.5	0.175±0.008	0.985	0.230±0.005	0.996	0.214±0.025	0.918

Table S5. Inactivation kinetic constants of *E. coli*, Total Coliforms, *Enterococcus* spp and *Pseudomonas* spp in UWW

[PMS] (mmol/L)	*E. coli* k (min^{-1})	*E. coli* R^2	*Total Coliforms* k (min^{-1})	*Total Coliforms* R^2	*Enterococcus* spp k (min^{-1})	*Enterococcus* spp R^2	*Pseudomonas* spp k (min^{-1})	*Pseudomonas* spp R^2
**UV-C (Figure 2a)**								
0	0.193±0.023<br>0.003±0.000	0.900	0.202±0.031<br>0.007±0.001	0.841<br>0.876	0.271±0.001	0.999	0.124±0.027	0.691
0.1	0.392±0.091<br>0.035±0.015	0.815<br>0.681	0.390±0.055<br>0.026±0.005	0.926<br>0.937	0.311±0.035	0.952	0.686±0.081<br>0.085±0.017	0.959<br>0.926
0.5	0.464±0.046<br>0.077±0.012	0.952<br>0.954	0.416±0.045<br>0.015±0.002	0.933<br>0.938	0.379±0.062	0.860	1.075±0.083	0.976
0.75	0.519±0.084<br>0.035±0.008	0.902<br>0.889	0.518±0.096<br>0.071±0.017	0.876<br>0.887	0.485±0.032	0.979	0.624±0.068	0.944
1	0.416±0.076<br>0.007±0.003	0.851<br>0.786	0.896±0.082<br>0.080±0.024	0.975<br>0.766	0.530±0.053	0.952	0.958±0.056	0.987

Table S6. Pseudo-first order degradation kinetic constants of Trimethoprim (TMP), Sulfamethoxazole (SMX) and Diclofenac (DCF) in UWW

[PMS] (mmol/L)	TMP		SMX		DCF	
	k (min^{-1})	R^2	k (min^{-1})	R^2	k (min^{-1})	R^2
**UV-C (Figure 2b)**						
0	0.001±0.000	0.957	0.071±0.004	0.968	0.110±0.018	0.903
0.1	NA		0.088±0.004	0.974	0.119±0.013	0.925
0.5	NA		0.076±0.003	0.986	0.205±0.040	0.779
0.75	0.033±0.002	0.935	0.144±0.004	0.993	0.197±0.018	0.943
1	0.045±0.002	0.974	0.185±0.010	0.970	0.314±0.039	0.900

NA= no fitting;

Table S7. ARGs means and standard deviation of the ratio detected in this study.

	16s rRNA	***intI1***	***sul1***	***$bla_{CTX-M32}$***	***bla_{TEM}***	***tetM***	***qnrS***
**Time (min)**	Mean±SD	Mean±SD	Mean±SD	Mean±SD	Mean±SD	Mean±SD	Mean±SD
**UV-C (Figure 3b)**							
0	1.00±0.01	0.67±0.01	0.52±0.00	0.38±0.01	0.48±0.00	0.51±0.01	0.40±0.00
20	0.87±0.01	0.51±.0.00	0.49±0.01	0.36±0.01	0.45±0.01	0.37±0.00	0.37±0.01
40	0.73±0.01	0.45±0.00	0.44±0.00	QL	0.39±0.00	0.35±0.00	QL
60	0.67±0.01	0.41±0.00	0.40±0.00	QL	0.39±0.00	0.36±0.00	QL
**PMS/UV-C (Figure 3b)**							
0	1.00±0.04	0.64±0.02	0.54±0.02	0.44±0.01	0.41±0.01	0.38±0.01	0.37±0.01
20	0.64±0.02	0.49±0.02	0.42±0.01	0.31±0.01	0.34±0.01	QL	QL
40	0.63±0.02	0.41±0.02	0.41±0.01	0.32±0.01	0.32±0.01	QL	QL
60	0.52±0.02	0.35±0.01	QL	QL	QL	QL	QL

QL, quantification limit

Table S8. Treatment efficiencies achieved in continuous flow (after 2 min) and in batch mode (considering the treatment time to achieve *E. coli* ≤ 10 CFU/100 mL and 80% of removal of CECs) by UV-C and PMS/UV-C (0.5 mmol/L).

		**Continuous Flow**		**Batch mode *E. coli* ≤ 10 CFU/100 mL 80% of removal of CECs**	
		**Time (min)**	**Log (C_i/C_f)**	**Time (min)**	**Log (C_i/C_f)**
E. coli	UV-C	2	0.5	30	4.6
	PMS/UV-C	2	1.6	15	4.7
CECs	UV-C	2	0.009	180	0.6
	PMS/UV-C	2	0.119	12	0.7

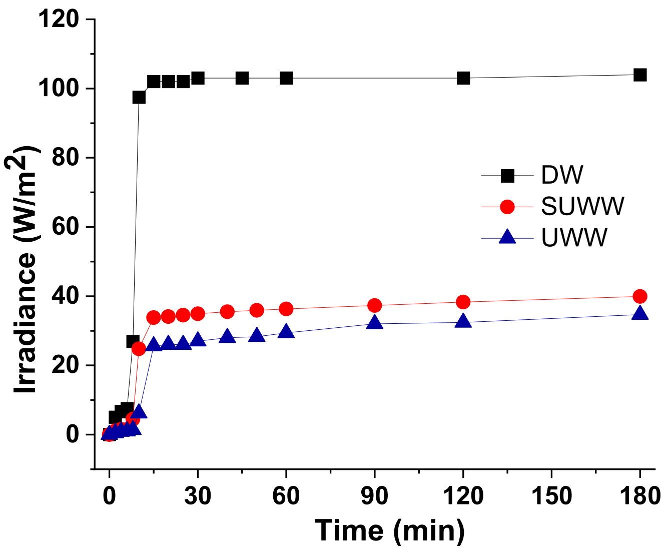

Figure S1. UV-C lamp irradiance (W/m^2) profiles registered in demineralized water (as baseline), SUWW and UWW.

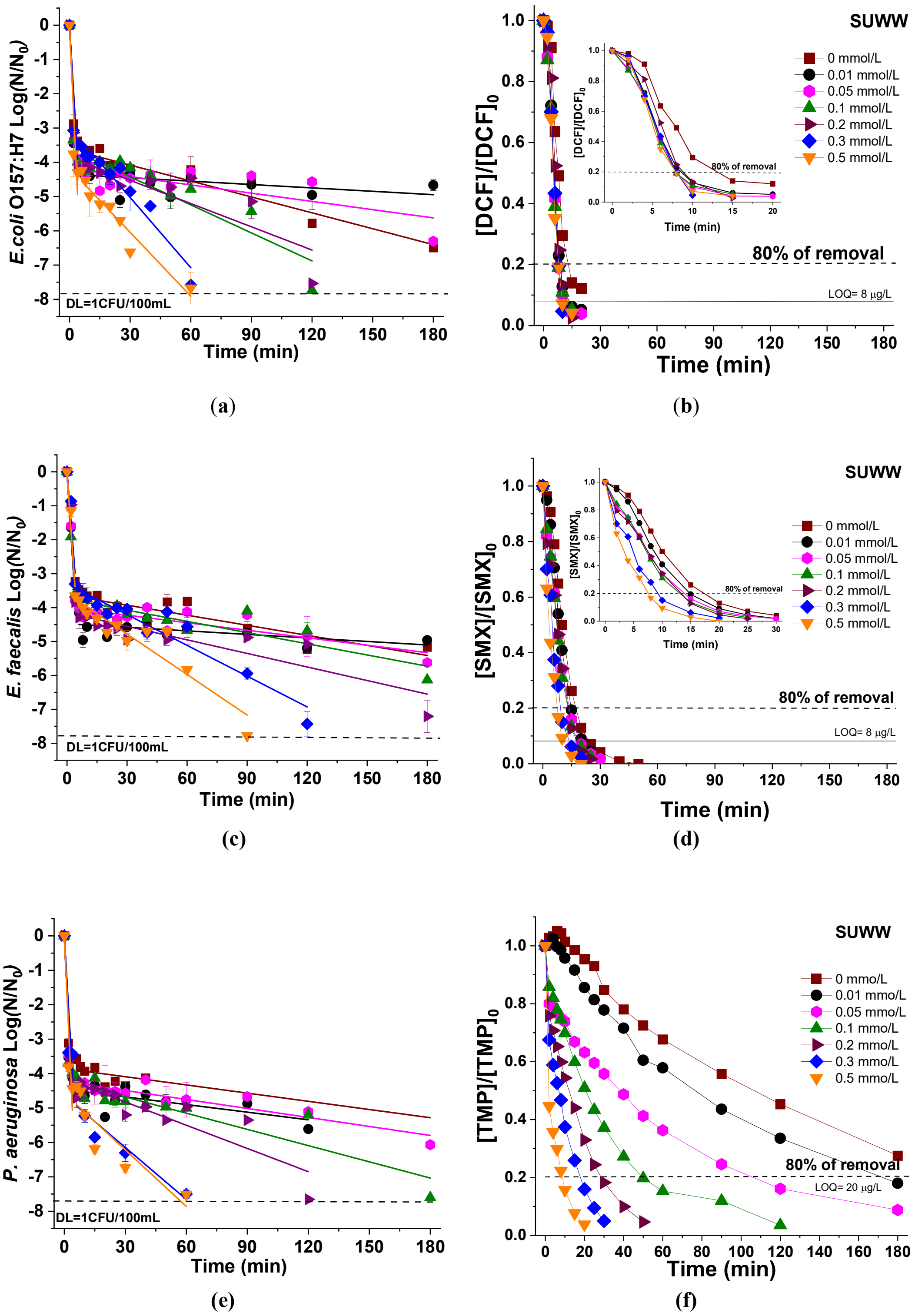

Figure S2. Inactivation profiles of *E. coli* (a), *E. faecalis* (c) and *P. aeruginosa* (e) and degradation profiles of DCF (b), SMX (d) and TMP (f) in the presence of increasing concentrations of PMS (0-0.5 mmol/L) and under UV-C irradiation in SUWW. Inserts: Enlargement of degradation curves.

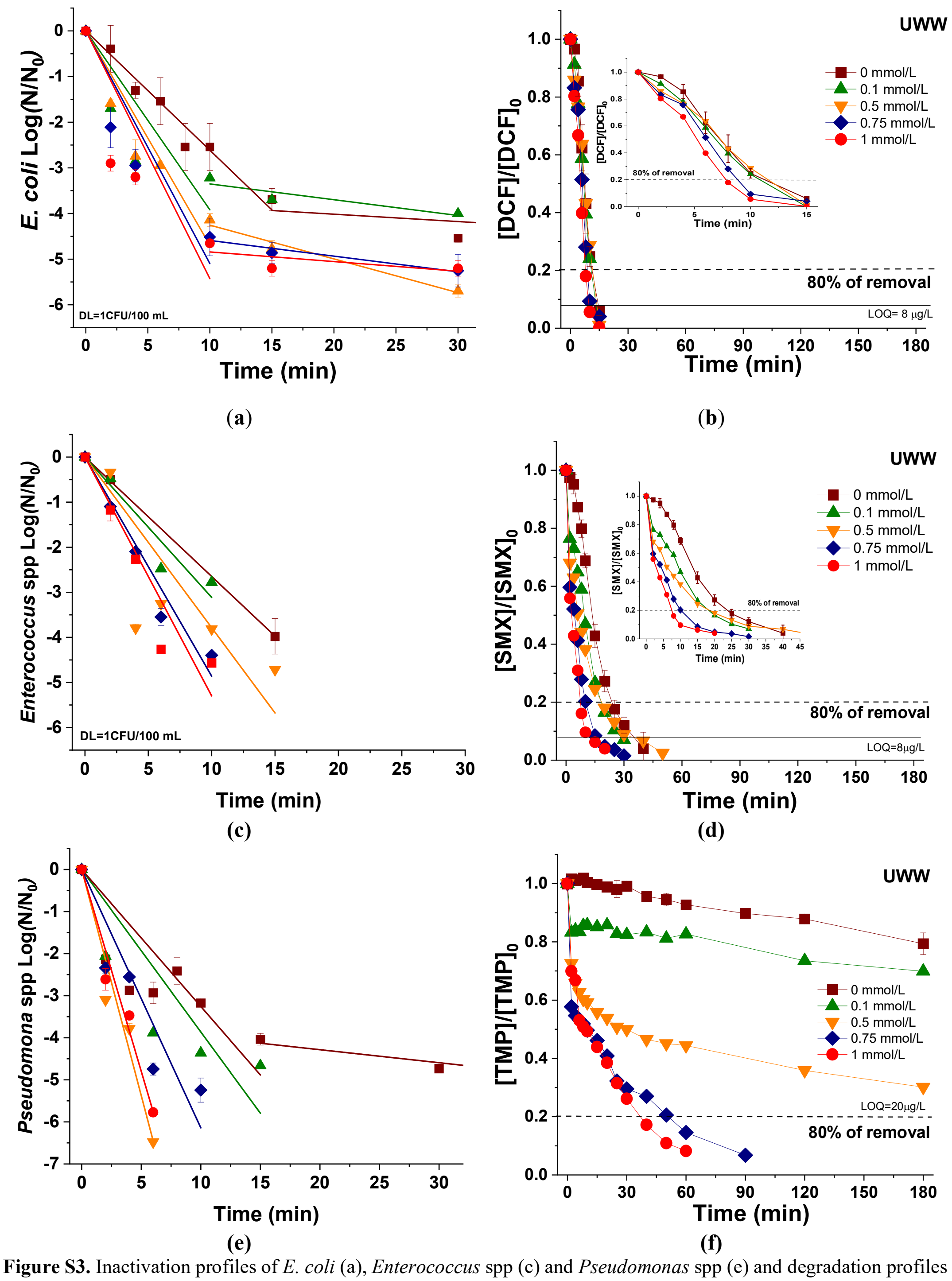

Figure S3. Inactivation profiles of *E. coli* (a), *Enterococcus* spp (c) and *Pseudomonas* spp (e) and degradation profiles of DCF (b), SMX (d) and TMP (f) in the presence of increasing concentrations of PMS (0-1 mmol/L) and under UV-C irradiation in UWW. Inserts: Enlargement of degradation curves.

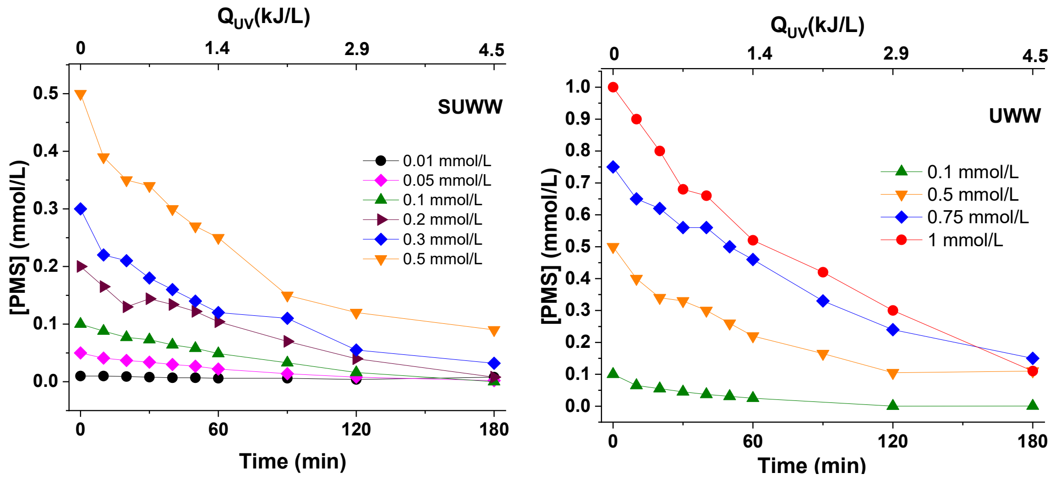

(a) (b)

Figure S4. PMS concentration decrease in function of time (minutes) and accumulative UV energy Q_{UV} (kJ/L) under UV-C treatment at different PMS concentrations in SUWW (0.01-0.5 mmol/L) (a) and in UWW (0.1-1 mmol/L) (b).
